# Clinical outcome of dynamic hip locking plates and proximal femoral nails anti-rotation-Asia for treating intertrochanteric femur fracture with lateral wall fractures in the elder patients

**DOI:** 10.18632/oncotarget.19754

**Published:** 2017-07-31

**Authors:** Hui Xie, Zhan Wang, Junji Zhang, Langhai Xu, Bao Chen

**Affiliations:** ^1^ Department of Orthopaedics, Jiaxing No. 2 Hospital, Jiaxing University, Jiaxing 314001, China; ^2^ Centre for Orthopaedic Research, Orthopedics Research Institute of Zhejiang University, Department of Orthopaedics, The Second Affiliated Hospital, School of Medicine, Zhejiang University, Hangzhou 310000, China; ^3^ Department of Infection Management, Suzhou Municipal Hospital, Suzhou 215002, China

**Keywords:** IFFs (intertrochanteric femur fractures), lateral wall fracture, DHLP (dynamic hip locking plates), PFNA-IIs (proximal femoral nails anti-rotation-Asia)

## Abstract

**Purpose:**

To compare the clinical results of DHLP (Dynamic hip locking plates) and PFNA-IIs (proximal femoral nails anti-rotation-Asia) for treating intertrochanteric femur fracture (IFF) with lateral wall fractures in the elder patients and provide a rationale for the clinical practice.

**Methods:**

A retrospective analysis of 43 patients of IFF with lateral wall fractures was performed from December 2009 to April 2015. Intraoperative variables and postoperative complications and function were compared between the two groups.

**Results:**

17 cases were treated by DHLPs, and 26 treated by PFNA-IIs. Patients were followed up from 6 to 16 months with an average of 11 months. Both the groups were comparable for demographic data before surgery. The PFNA-II group had less operation time, time of full weight bearing and healing time of fracture, but larger blood loss in comparison with the DHLP group (*p*<0.05). Additionally, internal fixation failure was significantly more in the DHLP group than in the PFNA-II group. The mean HHS and the rate of good-to-excellent in the PFNA-II group was significantly higher than that in the DHLP group both in third month after surgery (*p*<0.05).

**Conclusions:**

PFNA-IIs treatment should be recommended for the elderly patients of IFF with lateral wall fractures, because of its shorter operation time, faster full weight bearing, faster function recovery, and lower failure rate. However, more attention should be payed to its larger blood loss.

## INTRODUCTION

The unstable intertrochanteric femur fracture (IFFs) is a major orthopaedic challenge, and associated with high rates of complications and poor prognosis [1, 2]. Intramedullary and extramedullary fixation methods are commonly used for treating unstable IFFs [3, 4]. Currently, a variety of implants of internal fixation have been employed for unstable intertrochanteric femur fractures include proximal femoral locking compression plate (PFLCP), PFNA (proximal femoral nails anti-rotation), InterTan nail, Dynamic Hip Screw (DHS), et al [5–7]. However, the optimal management of unstable IFFs still remains controversial. Gotfried Y first emphasized the importance of lateral trochanteric wall and certainly suggested that an intact lateral trochanteric wall played a key role in the stabilization of unstable IFFs [8]. Subsequently, Palm H et al showed that a postoperative fracture of the lateral wall was closely related with a reoperation after an intertrochanteric fracture and pointed out intertrochanteric fractures should be classified according to the integrity of the lateral wall, especially in trials comparing fracture implants. Moreover, IFF with lateral wall fractures are a challenge for orthopedic surgeons. The optimal internal fixation for treating this type of unstable intertrochanteric fractures remains controversial. Thus, this study aimed to compare DHLPs with PFNA-IIs in the management of IFF with lateral wall fractures.

## RESULTS

**Figure 1:**
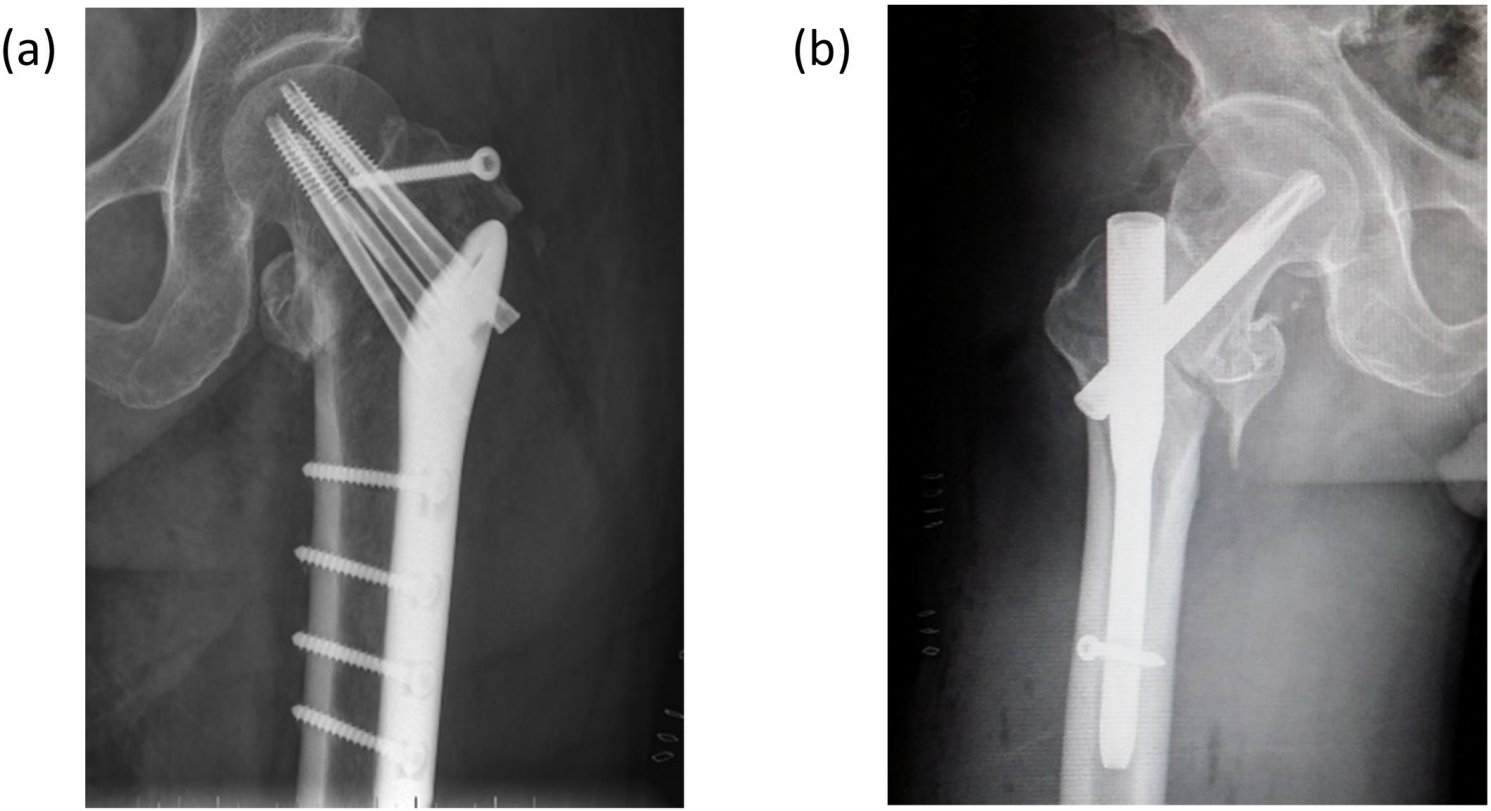
**(a)** patient treated with DHLP fixation device; **(b)** patient treated with PFNA-II fixation device.

**Figure 2:**
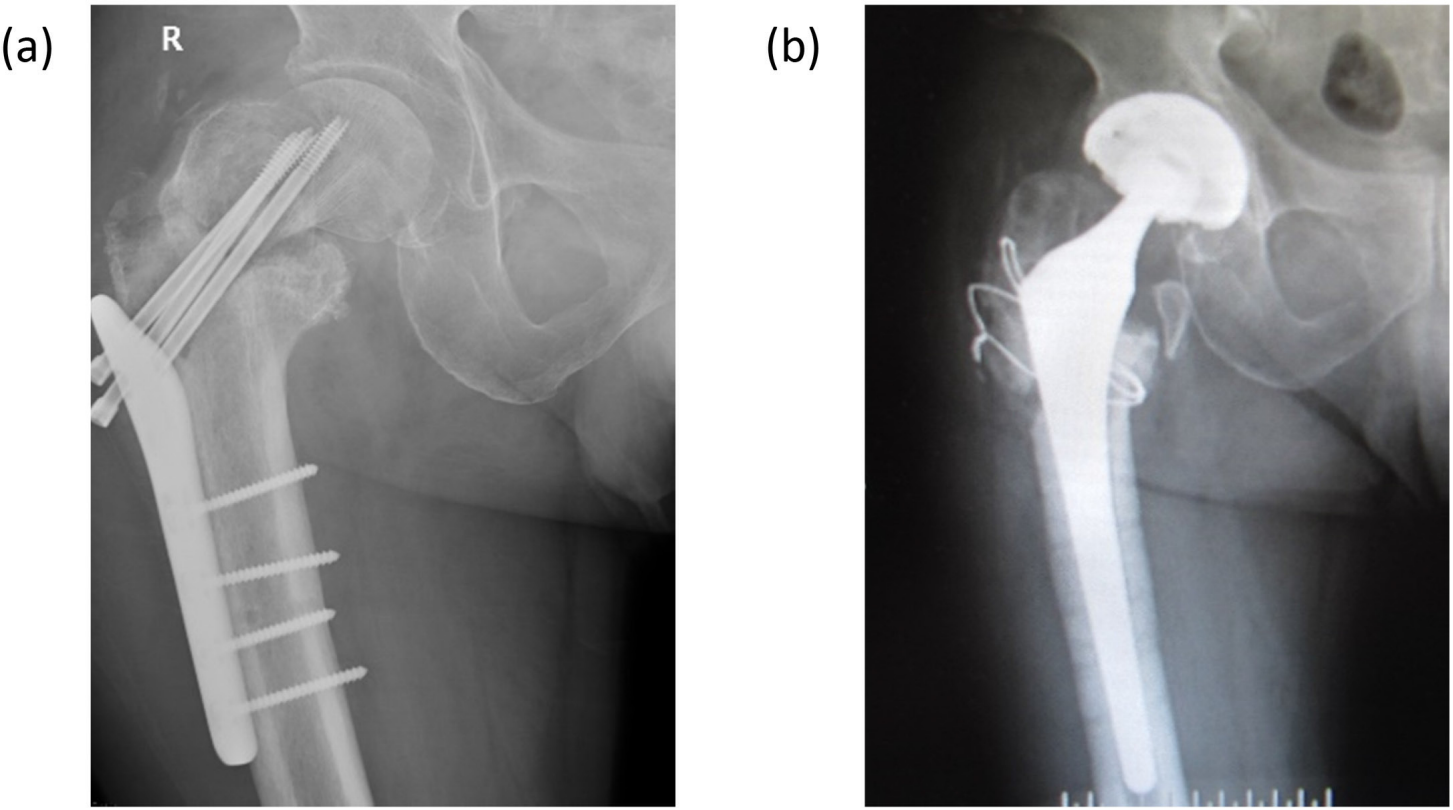
**(a)** Aggravating hip varus, withdrawal of screw, and no sign of fracture healing at nearly four months postoperatively; **(b)** Removal of internal fixation and total hip arthroplasty.

19 were male, 24 were female and the average age was 77 years old (ranging between 65 and 93). 17 patients were treated with DHLP fixation device and 26 patients were treated with PFNA-II fixation device (Figure 1a, 1b). The mean follow-up period was 11 months (ranging from 6 to16 months). Both the groups were comparable for demographic data before surgery (Table 1). The PFNA-II group had less operation time, time of full weight bearing and healing time of fracture in comparison with the DHLP group (*p*<0.05). Compared with the DHLP group, the PFNA-II group had larger blood loss (*p*<0.05). Internal fixation failure was significantly more in the DHLP group than in the PFNA-II group (Table 2). The failure type of these four failure cases were internal fixation screw loosening or withdraw. Two of four internal fixation failure in the DHLP group received delayed mobilization. The other two patients required revision. But only one finally received total hip joint replacement (Figure 2a, 2b) and one was lost during follow-up.

**Table 1:** Comparison of characteristics of the patients before surgery in two groups

Group	Number	Age(years)	Sex (number)		Fractured side (n)		Time for preoperative preparation
			Male	Female	Left	Right	
DHLP	17	75.77±7.22	8	9	6	11	5.41±3.57
PFNA-II	26	77.58±6.95	11	15	10	16	4.50±1.21
*P* value		0.415	0.76		0.83		0.702

**Table 2:** Comparison of intraoperative variables and clinical results after surgery in two groups

Group	Number	Operation time(mins)	Total blood loss(ml)	Time of full weight bearing (w)	Healing time of fracture(w)	Internal fixation failure (n)	Revision demand (n)
DHLP	17	64.19±11.30	525.88±56.69	12.88±2.00	14.53±2.03	4	2
PFNA-II	26	45.35±6.81	711.15±63.71	8.04±1.34	11.73±1.56	0	0
*P* value		0.00	0.00	0.00	0.00	0.02	0.15

The results of HHS after surgery were shown in (Tables 3 and 4). The mean HHS in the PFNA-II group was significantly higher than that in the DHLP group both in third month after surgery (*p*<0.05). Additionally, the rate of good-to-excellent in the PFNA-II group was also significantly higher than that in the DHLP group in third month after surgery (*p*<0.05). However, no statistically significant difference was found in the rate of good-to-excellent between two groups in sixth month after surgery (*p*<0.05).

**Table 3:** Comparison of HHS in third month after surgery between two groups

Group	Excellent (90–100 points)	Good (80–90 points)	Fair (70–79 points)	Poor (<70 points)	Good-to-excellent (%)	Mean in points
DHLP	3	4	6	3	43.75	75.25±11.23
PFNA-II	5	15	5	1	76.92	83.96±7.78
*P* value					0.03	0.01

**Table 4:** Comparison of HHS in sixth month after surgery between two groups

Group	Excellent (90–100 points)	Good (80–90 points)	Fair (70–79 points)	Poor (<70 points)	Good-to-excellent (%)	Mean in points
DHLP	3	7	4	2	62.50	81.94±8.08
PFNA-II	5	16	4	1	80.76	85.19±6.49
*P* value					0.28	0.16

## DISCUSSION

Management of unstable IFFs requires stable fixation that allows early mobilization and remains a challenge to orthopaedic surgeons. Recently, lateral wall reconstruction is seen as an important component in stabilization and fixation of unstable IFFs by providing a lateral buttress for the proximal fragment. The fracture of lateral wall can lead to collapse, which is a major cause of postoperative morbidity [8]. So, intertrochanteric fractures should be classified according to the integrity of the lateral wall. The study was initiated to compare PFNA-II and DHLP for differences in outcomes of IFF with lateral wall fractures.

IFF with lateral wall fractures represent a challenge for internal fixation. Palm et al [9]thought that a sliding compression hip screw was not sufficient for treatment of fractures involving the lateral wall and more methods should be needed to manage this condition. Using sliding hip screw in fractures with broken lateral wall could result in collapse, limb length shortening and poorer functional outcome [10, 11]. Gupta RK et al showed that lateral wall reconstruction using a trochanteric stabilising plate (TSP) in combination with a dynamic hip screw (DHS) can be successful [12]. Proximal femoral nail anti-rotation (PFNA) and locking compression plate (LCP) have good effectiveness in the treatment of intertrochanteric fractures with the lateral unsubstantial femoral wall in the elderly patients. Each has its own advantages and disadvantages [7]. However, Haq RU et al found that PFN (proximal femoral nail) was a better implant than reverse-DFLCP (reverse distal femoral locking compression plate) for IFFs with compromised lateral wall because of favourable intraoperative variables, better functional outcome and lower failure rates [13]. Additionally, Hu et al [14] thought that anatomic locking plate could be used for IFF with lateral wall fractures especially for severe comminuted fractures, difficult for intramedullary nailing to avoid re-injury of lateral wall.

Currently, there is no consensus regarding which type of internal fixation is the better option for unstable IFFs especially for IFF with lateral wall fractures. In our study, the PFNA-II group had less operation time, time of full weight bearing and healing time of fracture in comparison with the DHLP group. Moreover, internal fixation failure was significantly more in the DHLP group than in the PFNA-II group. Regarding functional outcomes, the mean HHS and the rate of good-to-excellent in the PFNA-II group was significantly higher than that in the DHLP group in third month after surgery. But no significant difference was observed in the mean HHS and the rate of good-to-excellent in the sixth month between two groups. Thus, PFNA-II is a more suitable option for early rehabilitation in patients with IFF with lateral wall fractures. Additionally, compared with the DHLP group, the PFNA-II group had larger blood loss (*p*<0.05). So, more attention should be paid on bleeding when performing PFNA-II for IFF with lateral wall fractures. To conclude, PFNA-II is more effective than DHLP in internal fixation of IFF with lateral wall fractures and can reduce complications and improve clinical outcomes.

## MATERIALS AND METHODS

Between December 2009 and March 2015, 43 elder patients of IFF with lateral wall fracture (AO/OTA type-31-A2, A3) operated at the Second Affiliated Hospital, School of Medicine, Zhejiang University, were investigated and completely followed up. These cases were performed by senior doctors in one team. DHLP fixation devices were from Tianjin Walkman Biomaterial Company Limited, China. PFNA-II fixation devices were from Trauson Medical Instrument Company Limited, China. Detailed clinical and radiological examination were performed on all patients. Demographic characteristics of the patients before surgery were compared between two groups. Fracture healing was assessed by X-ray reexamination. The function of the hip joint was assessed according to the Harris Hip Score (HHS). Out of a total of 100 points, 100 to 90 points were rated excellent; 89 to 80, good; 79 to 70, fair; and less than 70, poor [15, 16]. One patient in DHLP group did not receive further treatment after internal fixation failure. So, this patient was not included in the comparison of HHS after surgery.

The operation time, total blood loss [17], time of full weight bearing, healing time of fracture, number of internal fixation failure, number of revision and HHS in third and sixth month after surgery were compared between the two groups. The operation time refers to the duration from skin incision to skin suture (minutes).

The statistical analysis was performed using SPSS 23.0 software for all statistical analyses. Data were expressed as Mean ± SD (standard deviation). Student t test was used for quantitative variables between two groups. Categorical variables were analysed by the chi-square test where appropriate. *P*<0.05 was taken as significant. Under varied distributional conditions, Wilcoxon rank sum test was used for time for preoperative preparation and time of full weight bearing between two groups. Fisher exact test was used for internal fixation failure, revision and good-to-excellent between two groups.
